# Study of Correlation between Structure and Shape-Memory Effect/Drug-Release Profile of Polyurethane/Hydroxyapatite Composites for Antibacterial Implants

**DOI:** 10.3390/polym15040938

**Published:** 2023-02-14

**Authors:** Monika Bil, Magdalena Jurczyk-Kowalska, Kamil Kopeć, Marcin Heljak

**Affiliations:** 1 Centre for Advanced Materials and Technologies CEZAMAT, Warsaw University of Technology, Poleczki 19 Street, 02-822 Warsaw, Poland; 2 Faculty of Materials Science and Engineering, Warsaw University of Technology, Woloska 141, 02-507 Warsaw, Poland; 3 Department of Biotechnology and Bioprocess Engineering, Faculty of Chemical and Process Engineering, Warsaw University of Technology, Waryńskiego 1, 00-645 Warsaw, Poland

**Keywords:** smart materials, shape memory, composite’s structure, antibacterial properties

## Abstract

The effectiveness of multifunctional composites that combine a shape-memory polyurethane (PU) matrix with hydroxyapatite (HA) as a bioactive agent and antibiotics molecules results from a specific composite structure. In this study, structure-function correlations of PU-based composites consisting of 3, 5, and 10 (wt%) of HA and (5 wt%) of gentamicin sulfate (GeS) as a model drug were investigated. The performed analysis revealed that increasing HA content up to 5 wt% enhanced hydrogen-bonding interaction within the soft segments of the PU. Differential-scanning-calorimetry (DSC) analysis confirmed the semi-crystalline structure of the composites. Hydroxyapatite enhanced thermal stability was confirmed by thermogravimetric analysis (TGA), and the water contact angle evaluated hydrophilicity. The shape-recovery coefficient (R_r_) measured in water, decreased from 94% for the PU to 86% for the PU/GeS sample and to 88–91% for the PU/HA/GeS composites. These values were positively correlated with hydrogen-bond interactions evaluated using the Fourier-transform-infrared (FTIR) spectroscopy. Additionally, it was found that the shape-recovery process initiates drug release. After shape recovery, the drug concentration in water was 17 μg/mL for the PU/GeS sample and 33–47 μg/mL for the PU HA GeS composites. Antibacterial properties of developed composites were confirmed by the agar-diffusion test against Escherichia coli and Staphylococcus epidermidis.

## 1. Introduction

Multifunctional composites based on shape-memory polymers (SMPs) and bioactive ceramic fillers such as hydroxyapatite (HA) having drug-release functionality are attractive candidates for the development of implantable orthopedic devices with lower risk of post-surgery infections [1,2]. Implants with shape-memory properties can self-deploy in the implantation site and adjust to the specific shape of a tissue defect under natural triggers such as body temperature or the pH of a surrounding tissue [3]. This feature enables minimally invasive surgical procedures and helps avoid problems with implant fixing, thus allowing for pain reduction and the hastening of patient rehabilitation [4,5,6,7,8]. The drug-release functionality may address bacterial infections, thrombosis, osteomyelitis, periodontitis, and biomedical or inflammatory complications, which are the most common cause of implant failure [5,9,10,11,12]. HA particles incorporated into a polymer matrix may function as a reinforcing filler that improves the mechanical properties of polymer systems. Due to its osteoinductive properties, it induces a regenerative response in bone cells [13,14,15]. Therefore, a combination of SMP/HA composites and drug delivery enables to development of advanced implantable devices, with many advantages for the patient [16,17].

The shape-memory effect of thermoresponsive SMPs is associated with a specific morphology, which consists of flexible segments and permanent net points. The flexible/soft segments can undergo thermally reversible vitrification or crystallization transitions above a specific transition temperature (T_trans_), which could be either the glass-transition temperature (T_g_) or a melting temperature of the crystalline phase (T_m_). The soft segments are responsible for temporary shape fixation below T_trans_. The crosslinking net points or hard domains with T_g_ or T_m_ much higher than T_trans_ are responsible for the recovery of the permanent shape [5]. It is established that, to develop a multifunctional composite with a temperature-activated shape-memory effect and antibacterial functionality, the defined polymer morphology needs to be designed. The correlation between these functions should be understood and clearly defined [18,19].

Polyurethanes (PU) are extensively investigated as the thermoresponsive shape-memory polymers, due to their characteristic segmented structure and proven biocompatibility. Polyurethane properties can be easily tailored to specific requirements by modification of chemical composition. So far, various features of a polyurethane morphology, such as soft-segment structure, crosslinking density, and crystallinity degree, have been studied in the context of the shape-memory effect [18,20,21,22,23,24,25,26,27].

The effect of new functionality, such as local-drug delivery or bioactivity, provided by incorporating bioactive ceramic on previously established functions, is the second aspect considered in the literature when designing multifunctional materials [12,19]. It was reported by Christian Wischke et al. [28] that the loading of drugs into amorphous PU by the swelling of the polymer matrix did not disturb shape-memory functionality or T_trans_ of the material. Similarly, our study presented in previously reported work [29] showed that drug incorporation into semicrystalline, PU fibrous mats prepared through electrospinning did not affect the crystallinity, T_m_, or shape-memory properties of the investigated materials. On the other hand, the published results on the influence of ceramic nanoparticles on shape-memory properties are ambiguous. The shape memory properties depend on polymer type, the content of the ceramic filler and fabrication methods of the final material [30,31,32,33,34]. For example, in the case of PU/HA [31] and PLA/HA [30], it was found that increasing amounts of HA enhanced the fixity of temporary shape, due to additional interactions between functional groups of the polymer matrix and HA. Du et al. [35] reported that shape-memory properties of in-situ-prepared PLA/HA composites exhibited various shape-memory properties, depending on HAp content, with the best shape-memory effect for 15 wt%. Senatov et al. [36] revealed that 3D-printed PLA/HA scaffolds demonstrated higher stress recovery and T_trans_ than PDLA scaffolds, which make them unfavorable for medical applications. The nanocomposite with ten wt% of nano-HA showed the best shape-memory effect, but the further increase in HA resulted in a longer shape-recovery time.

Although many aspects of the polymer structure—shape-memory effect, or polymer structure—and drug-release-profile relationships were elucidated separately, the attempts to investigate the influence of composite structure on both these functions have not been well studied before. Moreover, there needs to be more data on the correlation between shape recovery and drug-release profile. Wischke et al. [28] compared drug-delivery profiles from polymer matrices before and after the shape-recovery process. This study analyzed the shape-memory properties of PUs/HA composites in a water environment and gentamicin-sulfate-release profile. The amount of hydrophilic drug released during the programming of shape recovery was studied from composites with different content of HA particles. The effect of hydroxyapatite content on the polymer structure was analyzed for correlation with drug delivery and shape-memory effect.

## 2. Materials and Methods

### 2.1. Materials

Biodegradable, segmented polyurethane (PU) consisting of 1,6-hexamethylene diisocyanate (HDI) and 1,4-butanediol (BD) as a hard segment and the mixture of D,L-lactide-co-glycolide (o-PLGA) and polycaprolactone diols (PCL) with various molecular weights was synthesized according to the method previously described [29]. HAp powder was obtained from Merck. Gentamicin sulfate (GeS), 1,6-hexamethylene diisocyanate (HDI), Tin (II)-2-ethylhexanoate (SnOct2), o-Phthalaldehyde (OPA), 2-mercaptoethanol, and sodium chloride were purchased from Sigma Aldrich and used as received. N, N-dimethylformamide (DMF) and BD were dried with a molecular sieve before use. Isopropanol, sodium borate, and methanol, were purchased from Chempur (Poland). *Escherichia coli* (PCM 2561, ATCC 8739) and *Staphylococcus epidermidis* (PCM 2118, ATCC 14990) strains were purchased from the Polish Collection of Microorganisms (Wroclaw, Poland). Cation-adjusted Mueller-Hinton Broth (CAMHB) and Mueller-Hinton Agar (MHA) were purchased from Merck KGaA (Darmstadt, Germany).

### 2.2. PU/HA/GeS-Composites Fabrication

The PU/HA/GeS composites consisted of 3, 5 and 10 wt% of HA and 5 wt% of GeS, according to PU weight. They were labeled as PU 3 HA GeS, PU 5 HA GeS, PU 10 HA GeS and PU 5 GeS (the sample containing GeS only). All the samples were prepared by solvent casting and the evaporation method. First, pre-weighted PU was dissolved in THF under stirring. Next, HA was dispersed in THF by a high-speed homogenizer for 30 min. Subsequently, the HA suspension in THF was slowly dropped into a polymer solution using a pipette, under continuous stirring. After 30 min. an appropriate amount of GeS powder was added to the solution and mixed for another 30 min. Next, the solution was poured on a Teflon mold, and the solvent was evaporated under a vacuum at 40 °C, to obtain films with a thickness of around 2 mm.

### 2.3. Characterization

The morphology of the surfaces of the composites were analyzed using SEM microscopy (Hitachi SU-8000, Japan), with an electron accelerating voltage of 5 to 10 kV. BSE and SE detectors were used in the research, which allowed for the analysis of the size and shape of the filler particles and their distribution in the PU structure. The samples were applied to a table covered with carbon tape and sputtered with a layer of gold. EDS analysis was performed with an electron accelerating voltage of 15 kV.

Infrared spectra of the chitosan microspheres and chitosan/polyurethane composites were collected in a Fourier-transform infrared spectrophotometer (Thermo Fisher Scientific model Nicolet 6700, Waltham, MA, USA). Measurements were carried out using the attenuated-total-reflectance (ATR) technique. Each sample was scanned 64 times, at a resolution of 4 cm^−1^ over the frequency range of 4000–400 cm^−1^. The hydrogen-bonding carbonyl ratio (Rt) was calculated as the ratio between the area below the hydrogen-bonding carbonyl band of urethane and the area of non-associated carbonyl band.

Raman spectra were recorded on Renishaw in a Via Raman Microscope using a 1200 L/mm grating and a 50× objective lens. A laser with 785 nm wavelength was used to excite the Raman signal. An integration time of 10 s was used, and 1 accumulation was taken for each spectrum. Raw data were processed with Wire 3.54 Renishaw software. 

DSC measurements were performed with differential scanning calorimeter DSC Q2000 (TA Instrument, New Castle, DE, USA) equipped with a refrigerated cooling system RCS90. All measurements were performed under a nitrogen atmosphere, using standard crimped-aluminum pans. The samples, weighing 2 mg on average, were scanned at a heating rate of 10 °C/min and a cooling rate of 5 °C/min in the temperature range of −85 ÷ 200 °C, using a heat/cool/heat cycle. Melting points (T_m_) were taken as the maximum in the endothermic peaks. The glass-transition temperatures (T_g_) were taken as the midpoint of the heat-flow curve pre- and post-transition. T_m,_ T_g_ temperatures and ΔH_m_ were determined from the second heating scan. Crystallinity χ_c_% was calculated according to the formula:

(1) $$\chi_{c}\%=\frac{{\Delta H}_{m}}{{(x}_{\mathrm{PCL}}\times {\Delta H}_{100\%})}\times 100\%$$

where ΔH_m_ and ΔH_100%_ are the heat of the fusion of the sample and the perfect crystal of PCL 135.44 J/g ΔH [29] and χ_PCL_ the weight fraction of PCL in the feed.

Thermogravimetric analysis was performed with TGA Q 5000 (TA Instruments, New Castle, DE, USA). Samples were analyzed under air atmosphere with a heating rate of 10 °C/min in the temperature range of 25 ÷ 800 °C.

The hydrophilic properties of the fabricated mats were tested on an OCA 20 goniometer (Data Physics, Filderstadt, Germany) using the sessile-drop method. In order to ensure adequate statistics, six measurements were used for each sample. Analysis of the surface allowed the determination of the water contact angle. 

### 2.4. Shape Memory

Two parameters were used to quantitatively describe the shape-memory abilities of the investigated SMPs: the recovery coefficient, *R_r_*, (describing the ability of the investigated SMP to return to its original shape) and the retention coefficient, *R_f_*, (describing preservation of the temporary shape of the investigated SMP specimen), given by the following formulas:

(2) $$R_{f}=\frac{\varepsilon_{u}}{\varepsilon_{m}}\;100\%\;(1)\;R_{r}=\frac{\varepsilon_{m}-\varepsilon_{k}}{\varepsilon_{m}}\;100\%$$

where *ε_u_* is the strain corresponding to the temporary shape (after load removal), *ε_m_* is the strain corresponding to the maximum length over which the specimen was stretched, and *ε_k_* is the strain of the specimen after the original shape-recovery phase is completed.

To identify the recovery coefficient, *R_r_*, and the retention coefficient, *R_f_*, a series of thermomechanical tests were performed using the TC-3 device from EBERS. The tests were carried out in uniaxial-tensile mode. Perpendicular specimens with a width of 6.3 mm and a base length of 15 mm were fabricated from previously cast films. The thermomechanical tests included three steps. In the first step, the specimen was heated up to 40 °C. The heating was conducted in a demineralized-water bath. The specimen was held at this temperature for 15 min; it was then stretched at constant-strain rate of 10%/min, up to 50% (*ε_m_*). In the second step, the specimen was rapidly cooled to 5 °C. The specimen was held at this temperature for 30 min. The cooling was conducted by filling the chamber with the mixture of water and ice. During the cooling phase, the shape of the investigated specimen was maintained and then the specimen was released from the grips. A marginal elastic recovery of the specimen would appear, and the *ε_u_* of the specimen was recorded. In the third step, the specimen was reheated to 37 °C, to recover its original shape. The temperature was kept for 30 min, and next, *ε_k_* of the investigated specimen was measured. The entire procedure was then repeated.

### 2.5. In Vitro Drug-Release Study

The GeS -release study was performed in phosphate-buffered saline (pH 7.4). PU/GeS and PU/HA/GeS composite films were placed in vials filled with 3 mL of a release medium. The vials were kept at 37 °C with rotational mixing of 50 rpm. At specified time points all release medium was withdrawn and replaced with the same amount of fresh buffer solution. The drug concentration was analyzed with an UV/VIS (Evolution 60, Thermo Fischer Scientific, Waltham, MA, USA) spectrophotometer at the wavelength of 332 nm, using OPA as derivatization reagent. The concentration of the drug in the specimen was determined from the standard curve. The mean values of 3 measurement ± standard deviations were reported. Several kinetic models were fitted with in vitro drug-release profiles, to understand the release kinetics.

### 2.6. Antibacterial Test

A total of 50 mL of CAMHB medium was inoculated with Escherichia coli or Staphylococcus epidermidis from MHA slants, and incubated at 37 °C for 18 h. Then, the bacterial suspensions were diluted with 0.9% (*w*:*v*) NaCl solution, to obtain a final suspension corresponding to 0.5 McFarland standard (1–2 × 108 CFU/mL). These suspensions were evenly spread on the surface of sterile MHA plates, using a cotton swab. After 15 min, samples of the tested materials in discs with a diameter of around 6 mm were gently and firmly placed on the agar surface. The resulting inhibition zones were measured after 24 h incubation at 37 °C (day 1). After day 1, the disks were transferred to new MHA plates inoculated with freshly prepared bacterial suspensions of the same density. The resulting inhibition zones were measured after 24 h incubation at 37 °C (day 2). The same procedure was repeated once more to measure the inhibition zones after another 24 h (day 3). Six replicates for each variant were tested (*n* = 6).

## 3. Results and Discussion

### 3.1. Characterization Results

**Figure 1:**
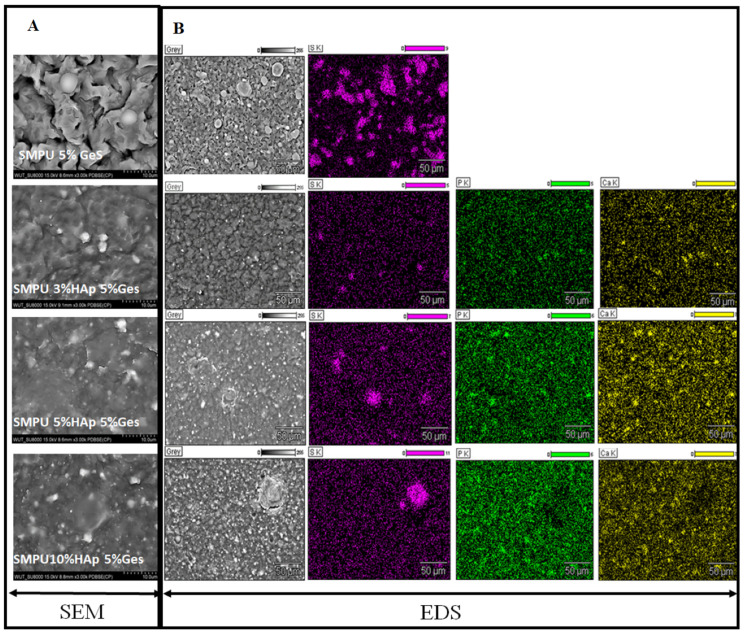
(**A**) SEM BSE images of composite surfaces with various contents of HA and 5 wt% of GeS. (**B**) EDS analysis showing distribution of S, Ca, P within PU matrix. Violet color—sulfur distribution originating from GeS, green—phosphor distribution from HA, yellow—calcium distribution that originates from HA.

The morphology of the composites was analyzed by SEM using the BSE mode to distinguish organic and inorganic phases in the composites. SEM micrographs of the composites are presented in Figure 1. SEM observations revealed that the PU 5% GeS sample showed the roughest surface, with clearly visible round-shaped and smooth inclusions. Micrographs of the PU/HA/GeS composites (Figure 1A) displayed the presence of white, irregular nanoparticles and the agglomerates with a diameter of up to few microns, uniformly distributed within the polymer matrix. Except for white irregular particles, light-grey and smoother regions are seen on SEM images of PU HA GeS composites (Figure 1A), similar to the PU 5 GeS, and these regions are well integrated with the polymer matrix. The EDS analysis confirmed that these smoother, light-grey inclusions are found in sulfur originating from GeS, while small, white, irregular-shaped particles are the HA phase containing Ca and P elements (Figure 1B).

**Figure 2:**
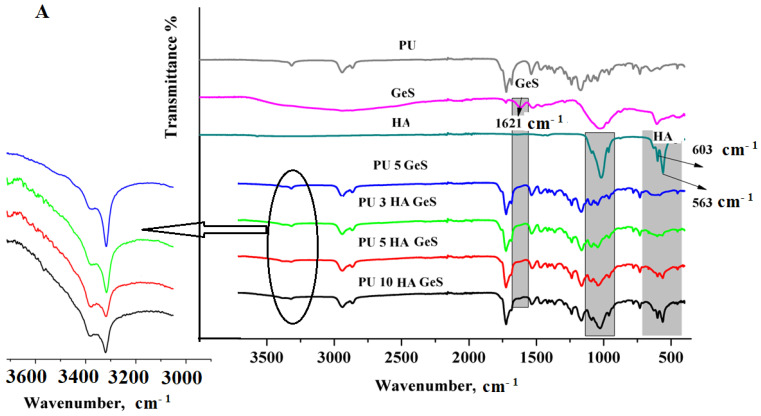

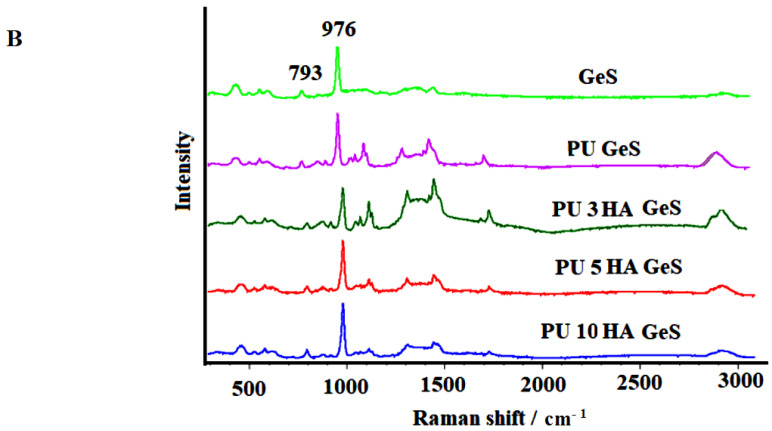
(**A**) FTIR–ATR spectra of PU, HA, GeS, PU GeS and PU HA GeS; (**B**) Raman Spectra of GeS and PU/HA composites with GeS.

FTIR–ATR analysis of the chemical structure of PU composites (Figure 2A) confirmed the presence of bands characteristic of polyurethanes associated with the hydrogen-bonded N-H-stretching vibration of the urethane group present at 3315–3321 cm^−1^. Additionally, peaks at 1728 cm^−1^ and 1685 cm^−1^ are assigned to the non-hydrogen bonded and the well-ordered, hydrogen-bonded C=O urethane band, respectively.

The amide-II bond correlated with combined N-H deformation and C-N-stretching vibrations was found at 1534–1538 cm^−1^ [1,2]. FTIR bands characteristic of HA were identified in the range of 560–601cm^−1^ shown in the spectra of PU/HA composites, which are associated with O–P–O vibrational modes. The intensity of these bands clearly increased with the higher content of HA nanoparticles in the composites (Figure 2A). Moreover, a new, strong band at 1027 cm^−1^ appeared in the spectra of PU-hydroxyapatite composites, due to the overlapping stretching vibrations of P−O present in the phosphate group of HA and the asymmetric-stretching vibration of SO4^2−^ of GeS. The additional weak absorption band found at the 3382 cm^−1^ and 1621 cm^−1^ wavenumbers corresponds to the amine- and amide-stretching vibrations of GeS, respectively [37]. 

The presence of GeS and its distribution in the polymer matrix were also confirmed, using Raman spectroscopy (Figure 2B). The most intensive bands of GeS were at 976 cm^−1^ of the Raman spectrum, with a less intense peak at 790 cm^−1^ (Figure 2B), which could be assigned to C−O−C-stretching and C−H-rocking vibrations, respectively [38].

The comparison of the Raman spectrum for GeS and PU GeS showed that the characteristic peaks of GeS were retained unchanged, which leads to a conclusion that there was no interaction between the drug and polymer functional groups. Only a slight shift in the GeS characteristic bands toward higher wavenumbers in the spectra of the PU/HA composites suggested that some interaction between GeS and HA occurred. However, no new bands appeared that could indicate the creation of new chemical bonds between GeS and HA.

**Figure 3:**
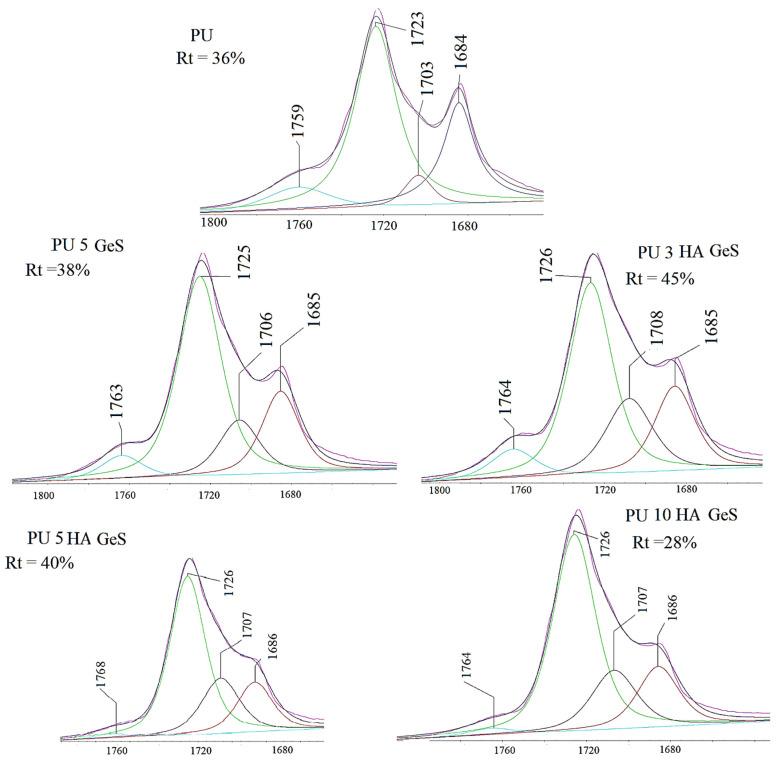
FTIR–ATR spectra of carbonyl region after deconvolution and Gaussian fitting. Rt—carbonyl hydrogen-bonding index.

After the deconvolution of the carbonyl band (Figure 3), additional peaks were found at 1706–1708 cm^−1^, which correlated with hydrogen-bonded urethane carbonyl in the disordered region, and 1759–1768 cm^−1^, assigned to the carbonyl groups of the PLGA segments. It is well known that hydrogen bonds act as physical crosslinking points that significantly influence polyurethane properties, including the shape-memory effect [39]. The hydrogen bonds can be formed between the NH of urethane and C=O urethane or C=O of the soft segments. The position of the peak related to N-H of urethane indicates that all N-H groups within urethane bonds are hydrogen bonded. To evaluate the amount of carbonyl groups that participate in hydrogen-bonding formation, the hydrogen-bonding carbonyl ratio (Rt) was calculate, and results are presented in Figure 3. The Rt value increased after the addition of 3% and 5% of HA, in comparison to PU GeS. However, the higher content of HA resulted in a decreasing value of Rt.

**Figure 4:**
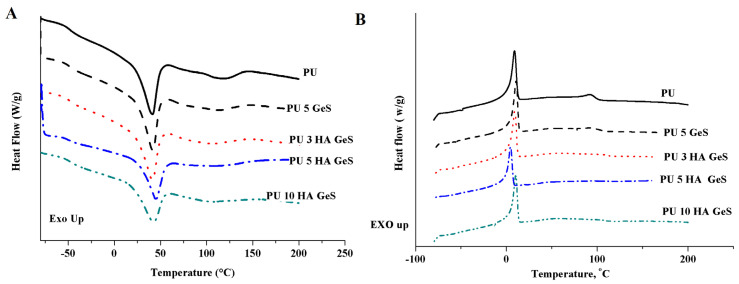
Thermal-analysis results: (**A**) DSC 2nd-heating scan; (**B**) DSC-cooling scan.

The highly hydrogen-bonded hard segments between the urethane bonds may act as physical cross-links that enhance shape-memory properties, but also may lead to a higher degree of phase separation between the hard and soft segments. The lower values of Rt for PU 10 HA GeS indicate that the higher content of HA interrupted the formation of hydrogen bonds within hard segments. Moreover, it is interesting to note that the intensity of the peak at 1706–1708 cm^−1^ assigned to hydrogen-bonded urethane carbonyl in the disordered region increased after loading of GeS and HA (Figure 3). These findings suggest that some hydrogen bonds could be formed within the interphase of the soft and hard segments, where the higher extent of the phase mixing could occur. These observations may be correlated with the wider melting peaks of the crystalline phase observed in Figure 4, which presents DSC thermograms.

The crystalline phase of these composites acts as the switching segments, and the melting temperature (T_m_) acts as the shape-memory switch. To evaluate the effect of GeS and HA content on crystallization ability and melting point, DSC analysis was performed, and obtained thermograms are presented in Figure 4A,B. The analysis results are summarized in Table 1. Thermograms presented in Figure 4A show that all samples are semicrystalline, which confirms the presence of a sharp endothermic peak with T_m1_ around 42 °C, resulting from the melting of the PCL crystalline fraction and the exothermic transition found (Figure 4B) around 5 °C, characteristic of the crystallization process. Additionally, the broad endothermic peak with T_m2_ around 100 °C was present in all thermograms. This broad peak indicates the presence of the heterogenous crystalline phase within hard segments or some imperfect crystals of PCL mixed with PLGA segments. The peak widening suggests the formation of imperfection within crystal regions, with crystallites of diverse sizes [40].

A more heterogeneous crystal structure arises from the hindered orientation of PCL segments due to the presence of HA particles and the mixing with PLGA segments. However, according to the results shown in Table 1, T_m1_ did not change, due to HA incorporation into the PU matrix, T_m2_ decreased after the incorporation of GeS, and additional decreasing was observed with an increasing amount of HA (Table 1). The crystallinity degree of PCL segments slightly decreased, from 24% for PU after the incorporation of GeS and HA, to the lowest value of 21% for PU 5 HA GEs (Table 1). 

**Figure 5:**
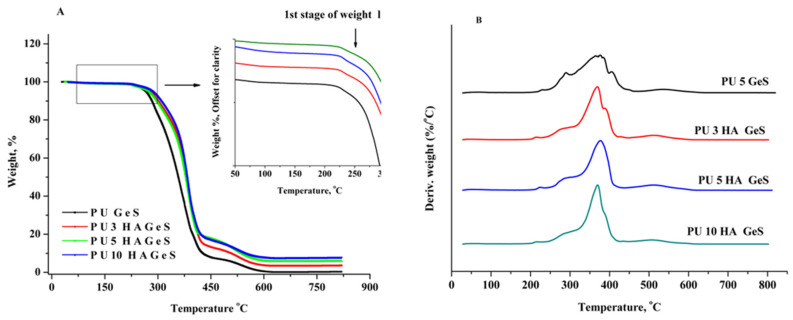
(**A**) TG and (**B**) DTG curves of PU composites, with GeS and various amounts of HA under air atmosphere. TG curves were shifted along Y-axis for clarity in the inset of Figure 5A and in Figure 5B.

These results show that the melting temperature of the crystalline phase of polymer macromolecules (T_m1_), which also acts as the trigger for the shape recovery, is affected by the structure of the polymer matrix, and the incorporation of bioactive ceramic did not change previously established features of the material. However, the broadening of the peak correlated with the melting of the crystalline phase of PCL (Figure 4A) segments, indicating that more heterogeneous imperfect crystals are formed with the increasing amount of HA. The TGA analysis presented in Figure 5 showed three steps of mass loss of PU correlated with the decomposition of various functional groups. The first stage of mass loss, which is clearly shown in the inset of Figure 5A, is correlated with the decomposition of GeS [41] and urethane bonds, and is associated with peaks I and II at T_max_, in the range of 227–233 °C and 289–297 °C, respectively, as shown on the DTG curve (Figure 5B). The next stage, correlated with the highest percent of mass lost, is attributed to the decomposition of ester bonds, with a maximum decomposition rate in the temperature range from 370 to 387 °C (Figure 5B), and the third stage, above 450 °C, is correlated with the pyrolysis of the degradation products [42,43]. The residual weight is consistent with the weight of HA incorporated into the polymer matrix (Table 2). Moreover, according to the results summarized in Table 2, thermal stability, evaluated as temperature for 5% of weight loss of the HA composites, increased in comparison to PU 5 GeS, which is consistent with the previously reported study [44].

### 3.2. Shape-Memory Results

**Figure 6:**
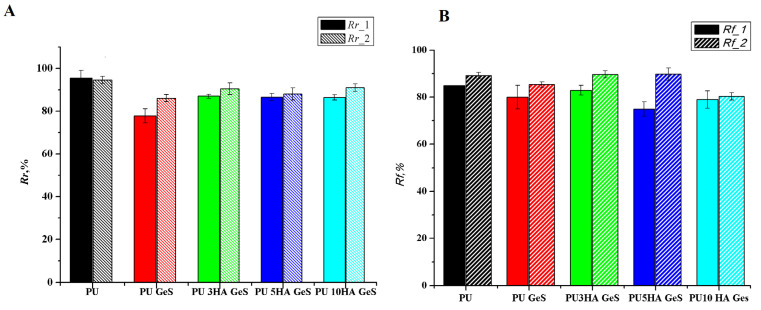
The shape-memory coefficients of (**A**) *R_r_* and (**B**) *R_f_*, calculated based on cyclic-thermomechanical-tensile test.

Quantitative assessment of the shape memory effect through strain-controlled shape-memory cycles showed good shape-memory properties for all materials. The results of *R_r_* and *R_f_* coefficients are presented in Figure 6A,B, respectively. The recovery coefficient, *R_r_*, of PU decreased after the addition of GeS and HA, from 94% for pristine PU to the range of 77–85% for PU/HA/GeS composites, after the first cycle. The lowest value of *R_r_* after the first cycle was found for PU GeS, and the highest for the composites with 3 wt% and 5 wt% of HA. These results correlate with the highest amount of highly ordered hydrogen bonds within hard segments, which were shown in the FTIR analysis (Figure 3). While some differences in shape recovery were noticed after the first cycle, after the second cycle of the shape-memory programming, the *R_r_* coefficient increased for all composites to within the range of 89–91%, and was comparable for all samples.

The enhancement of shape recover, *R_r_*, after the second cycle was previously reported [45,46], and related to macromolecule reorganization and lower chain entanglements within the polymer matrix after the first thermomechanical cycles. Additionally, the water may act as a specific plasticizer that enhances molecules’ lability, leading to more perfect crystals within the hard and soft segments, and finally improving the shape-memory effect after two cycles of programming.

The same trend was observed for the retention-shape coefficient. After the second cycle for the PU composites, the *R_f_* value was in the range of 80% for PU 10HA GeS and 89% for PU 3HA GeS, and these values are comparable to the Rf of the pristine-PU film (Figure 6B). The lowest *R_f_* coefficient after the second cycle was found for the composite with the highest content of HA. In the PU HA GeS composites, crystalline segments of PCL chains are responsible for temporary-shape fixation. These results show that adding ten wt% of HA nanoparticles may hinder macromolecule mobility and thus delay the crystallization process and formation of uniform crystals. The lower number of perfect crystals results in a lower number of physical net points able to create stronger interactions to fix the temporary shape. The results are in good agreement with the DSC results, where some broadening of the melting peak was observed (Figure 4A), with increasing HA content indicating the heterogeneous morphology of formed crystals.

### 3.3. Results of GeS-Release Study

**Figure 7:**
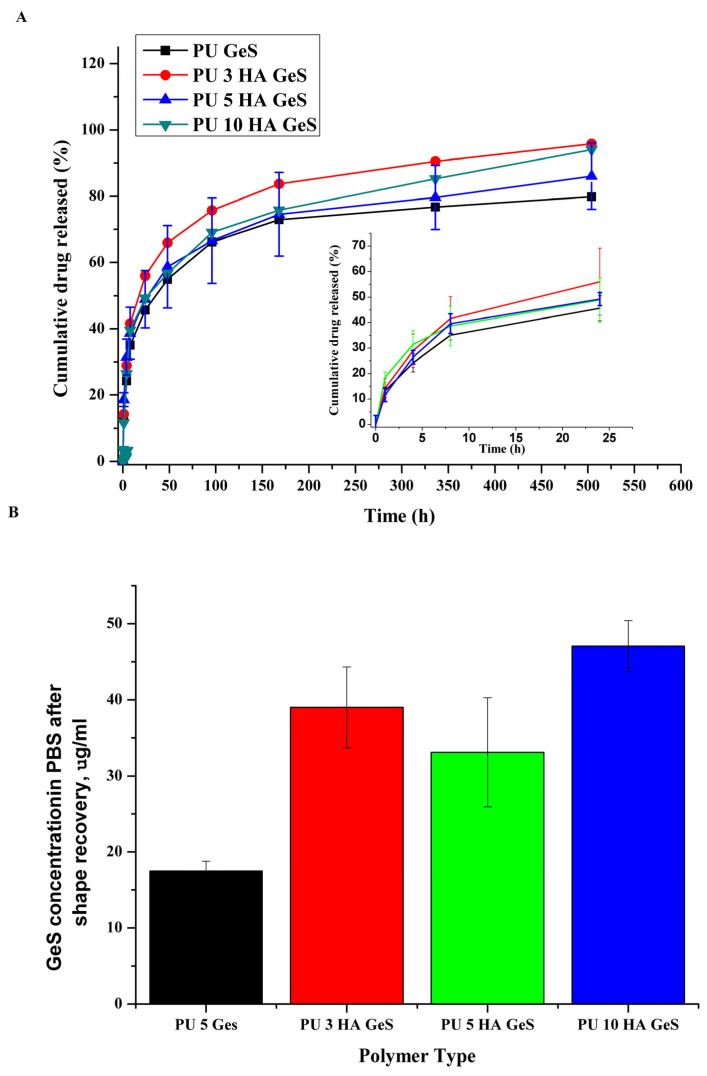
(**A**) Gentamicin-sulfate-release kinetics from PU/HA/GeS; (**B**) GeS concentration in water after shape recovery.

GeS was used as a model drug to evaluate the potential application of the PU/HA composite as a matrix for local-drug delivery. In order to evaluate the effect of composite structure on the GeS release profile, an in vitro study on PBS was conducted. As shown in Figure 7, two stages of the release profile were observed, with an initial burst release that reached around 40% of the total mass of GeS after 8h of release study from the PU and PU/HA films. Next, a sustained-release profile was observed for all samples, with the total cumulative mass of GeS released from PU films in the range of 83% for PU 5 GeS and 98% for PU 10 HA GeS. The initial burst-release stage is connected with the diffusion of GeS from the composite surfaces and the dissolution process [28,47].

Additionally, the analysis of the water after the shape-recovery process revealed the higher concentration of GeS released from the PU/HA composites in comparison to the PU/GeS sample (Figure 7B). These results indicate that the process of shape recovery itself, which is associated with the reorganization of macromolecules and the release of stress accumulated in the sample may be a driving force for the drug release. This is important from the application point of view, as an additional amount of drug released during the first minutes after implantation may enhance the burst-release effect observed in the drug-release study. This higher amount of drug delivered to the implantation site may provide a positive antibacterial effect.

In order to analyze the mechanism of GeS transport from the PU/GeS and PU/HA/GeS composites, in-vitro-release data were fitted to zero-order, first-order, Higuchi, and Korsmeyer–Peppas mathematical drug-release models [48]. The model that best fitted the release data was evaluated by the correlation coefficient (R^2^). R^2^ values for all formulations in various models are given in Table 3.

In the case of PU 5GeS, the best fitting was found for the Higuchi model with the R^2^ coefficient of 0.976, but also good linear fitting was found for the Korsmeyer–Peppas release model, with an R^2^ coefficient of 0.972 and diffusion exponent (n) of 0.37. The PU/HA/GeS composites follow the Korsmeyer–Peppas model (Table 3), with n values of 0.38 ± 0.01 for PU 3 HA GeS, 0.32 for PU 5 HA GeS and 0.42 ± 0.05 for PU 10 HA GeS (Table 3). These results show a pseudo-Fickian behavior of diffusion [49] for all tested materials. The obtained results revealed that the incorporation of HA into the PU matrix sustained its ability for sustained delivery of GeS, and did not affect the drug-release mechanism.

### 3.4. Antibacterial Test Results

**Figure 8:**
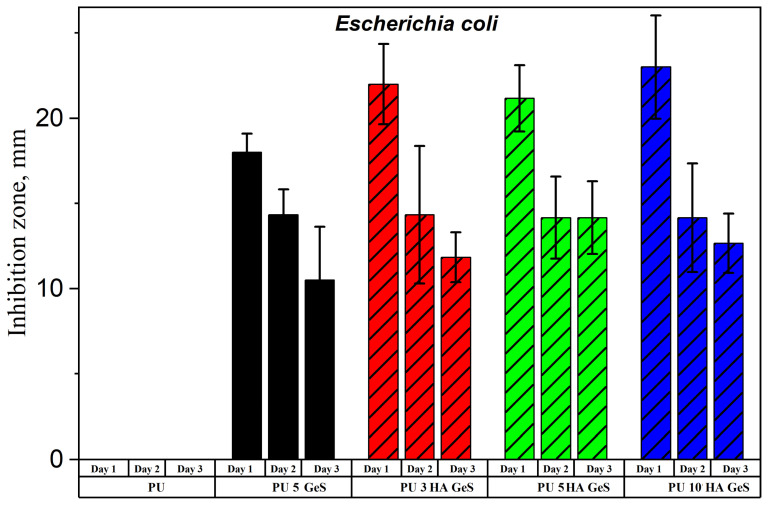

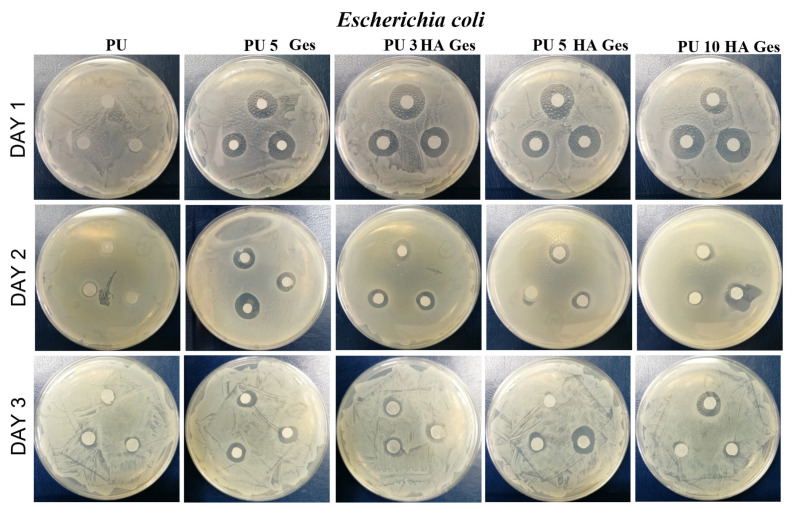
Inhibition zones of *E. coli* growth around nanocomposite-film discs.

**Figure 9:**
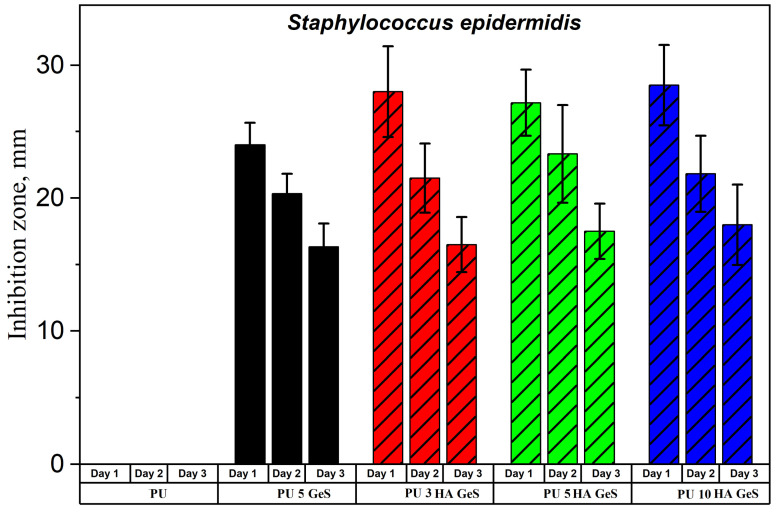

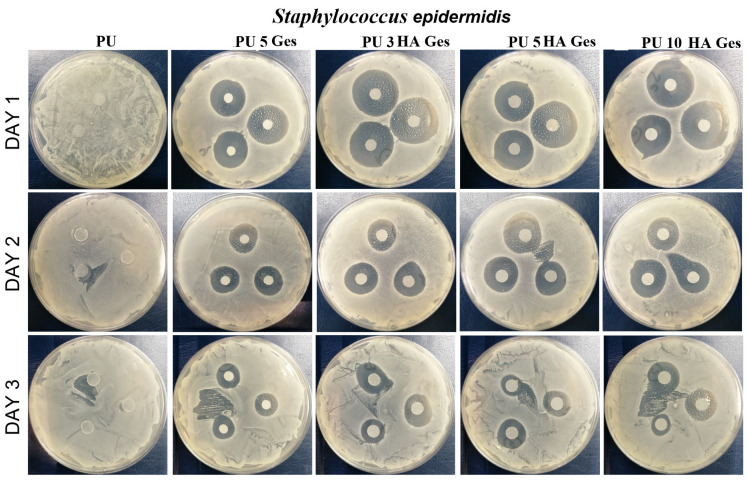
Inhibition zones of *S. epidermis* growth around nanocomposite-film discs.

In order to evaluate the anti-infection potential of PU/HA composites, antimicrobial tests were carried out using the disc-diffusion method against *E. coli* and *S. epidermidis*. *E. coli* and *S. epidermidis* were selected as model gram-negative and gram-positive strains, respectively. Both of these strains were originally isolated from the human body, and according to the literature reports, these pathogens are mainly responsible for post-implantable infections of orthopedic devices [49]. The inhibition zones of bacterial growth during three days of GeS diffusion from the PU/HA/GeS discs are presented in Figure 8 and Figure 9. The results of antibacterial tests carried out on the PU films (without drugs) have shown dense films of *E. coli* and *S. epidermidis* on the Petri plate, due to the lack of antibacterial activity of the PU samples without GeS. On the contrary, however, samples loaded with five wt% of GeS confirmed antibacterial effects against *E. coli* and *S. epidermidis* over three days of the diffusion test. After 24 h of GeS diffusion, the lowest diameter of the inhibition zone was found for the PU 5 GeS samples for both types of bacteria (Figure 8 and Figure 9). The inhibition zones for composites with HA were higher after 24 h diffusion for both tested bacteria, which correlates with the amount of GeS released (Figure 8A). Next, the gradual decline in the diameter of the inhibition zones for *S. epidermidis* was observed after the second and third days of incubation, for all tested samples. However, the zones of S. epidermidis inhibition around all HA composites had a 17–18 mm diameter after the third day of the diffusion test. The zones of *E. coli* inhibition were lower, with a diameter of 11–15 mm after the second and third days of diffusion. A slightly better antibacterial effect was observed for composites with HA; specifically, higher inhibition zones were observed around samples with 5 wt% of HA. These results show that GeS-loaded composites show antibacterial activity against most common bacteria responsible for infections after the implantation of orthopedic implants, and therefore are good candidates for developing drug-delivery medical devices.

## 4. Conclusions

Shape-memory properties, the release profile of GeS and antibacterial properties of PU/HA composites were evaluated, and correlated with the materials’ morphology and structure. The analysis of the impact of HA content on the structure and the resultant material properties revealed that the incorporation of HA in the range of 3–5 wt% enhances hydrogen-bonding interaction, which results in better shape recovery than for pristine PU with 5 wt% of GeS after the first thermomechanical cycle. Additionally, HA particles increased the thermal stability of the composites in comparison to pristine PU by 20 °C, and hydrophilicity by approximately 20°. Analysis of the drug concentration in water after the shape-recovery test showed that hydrophilicity changes resulted in a faster release of GeS during shape recovery. The T_m_ of the PCL-switching segments was not changed after HA and GeS loading, and was around 41 °C. Although GeS and HA incorporation affected the morphology of the crystalline phase, all PU HA GeS composites after the second cycle of the shape-memory programming had satisfactory shape-memory properties, with *R_r_* in the range of 89–91%. All composites showed antibacterial activity against *S. epidermis* and *E. coli* strains over three days of the agar-diffusion assay, which make these materials promising candidates for implantable drug-delivery devices. 

## Figures and Tables

**Table 1:** Summary of DSC and water-contact-angle results.

Sample	T_g_, °C	T_m1_ °C	ΔH_1_, J/g	χ_c_, %	T_m2_	ΔH_2_, J/g	Water Contact Angle °
PU	−48	41	22	24	115	10	93 ± 3.4
PU 5 GeS	−48	41	21	23	108	9	88 ± 3.2
PU 3 HA GeS	−48	42	20	22	101	9	74 ± 2.7
PU 5 HA GeS	−49	42	19	21	99	8	65 ± 3.8
PU 10 HA GeS	−50	41	21	23	97	8	61 ± 2.6

**Table 2:** The results of TGA analysis.

Sample	Peak I Max, °C	Peak II, °C	Peak III Max, °C	Peak IV, °C	T, Weight Loss 5%	Residual Weight, %
PU/GeS	227	289	370	404/535	266	0.3
PU 3HA/GeS	232	294	386	406/528	278	3.5
PU 5HA/GeS	232	295	385	520	274	5.7
PU 10 HA/GeS	233	297	387	522	285	8.5

**Table 3:** The correlation coefficient R^2^ from in-vitro-release data of GeS for different release-kinetic models.

Sample	Zero Order	1st Order	Higuchi	Korsmeyer–Peppas	
	Regression Co-Efficient R^2^ Value				Diffusion Exponent (*n*)
PU 5 GeS	0.856 ± 0.028	0.933 ± 0.008	0.976 ± 0.005	0.972 ± 0.014	0.37 ± 0.03
PU 3 HA GeS	0.616 ± 0.05	0.805 ± 0.04	0.861 ± 0.02	0.939 ± 0.04	0.38 ± 0.08
PU 5 HA GeS	0.680 ± 0.02	0.862 ± 0.008	0.871 ± 0.01	0.971 ± 0.012	0.32 ± 0.08
PU 10 HA GeS	0.694 ± 0.073	0.808 ± 0.09	0.871 ± 0.06	0.952 ± 0.027	0.42 ± 0.06

## Data Availability

The data presented in this study are available on request from the corresponding author.
